# Global perspectives and clinical trends in Qigong research: a bibliometric and visual analysis (2005–2025)

**DOI:** 10.3389/fmed.2026.1707980

**Published:** 2026-05-07

**Authors:** Zhouluo Wang, You You, Xice Zhang, Jingyu Wang, Yi Sun

**Affiliations:** 1 School of Philosophy and Sociology, Jilin University, Changchun, China; 2 College of Physical Education, Kookmin University, Seoul, Republic of Korea; 3 Department of Sport Leisure, Sungshin Women's University, Seoul, Republic of Korea; 4 Physical Education College, Jilin University, Changchun, China

**Keywords:** bibliometrics, clinical study, holistic health, primary care, Qigong, traditional Chinese medicine, visualization

## Abstract

**Objectives:**

Qigong, a mind–body practice of Traditional Chinese Medicine, has gained increasing recognition in global health and growing scientific attention. However, the global research landscape remains insufficiently mapped. Therefore, a bibliometric analysis is needed to evaluate research status, hotspots, and clinical trends, thereby guiding future academic inquiry and practical implementation.

**Methods:**

Publications on Qigong from 2005 to 2025 were retrieved from the Web of Science Core Collection and PubMed databases. Three visualization tools, R software, VOSviewer, and CiteSpace, were applied to analyze publication trends, countries, institutions, funding agencies, sources, references, keywords, and MeSH terms, encompassing descriptive indicators, network mapping, clustering, and burst detection.

**Results:**

Trends in annual publication outputs have shown steady growth with minor fluctuations from 2005 to 2025. China (*n* = 959, 55.4%) and the USA (*n* = 292, 16.9%) lead in Qigong research, with their institutions serving as central hubs for global academic collaboration, alongside their public funding agencies standing as the foremost contributors. *Medicine* (100 articles) and *Journal of Alternative and Complementary Medicine* (1,748 citations) rank among the most prolific and frequently cited sources. Research hotspots primarily focus on Qigong for cognitive and physical health in geriatric populations, disease management, and quality-of-life enhancement across the lifespan. MeSH term analysis of clinical studies reveals mental and behavioral disorders as the most investigated category, with depression, fatigue, anxiety, and pulmonary diseases among the predominant conditions examined. Among Qigong modalities, Baduanjin is the most extensively studied, followed by Liuzijue and Yijinjing. In clinical trends, recent developments reveal a trajectory toward disease diversification, modality specialization, and population expansion. Since 2023, an emerging frontier has focused on college students' physical and mental wellbeing, integrating technologies such as virtual reality into Qigong interventions to enhance motivation and adherence.

**Conclusion:**

Over the past two decades, global attention to Qigong has progressively expanded, marked by broad international participation alongside concentrated scholarly leadership, rooted in Qigong's cultural and historical foundations. Future investigations could further foster initiatives spanning multiple disciplines and cultures, bridging tradition and innovation to advance holistic health and wellbeing for all.

## 1 Introduction

For centuries, traditional medicine systems have played a significant role in global health, particularly in supporting household and community wellbeing (1). The Shanghai Declaration 2016 (2), the Declaration of Astana 2018 (3), and the Gujarat Declaration 2023 (4) have all emphasized the integration of traditional medicine into primary health care, which serves as a cornerstone for achieving health and wellbeing for all, with 170 World Health Organization (WHO) Member States reporting active use of such systems. Among these, Qigong, a practice of Traditional Chinese Medicine (TCM), has attracted growing attention for being accessible, low-risk, cost-effective, and sustainable in promoting both physical and mental health (5). Originating over 5,000 years ago, Qigong encompasses a wide variety of energy exercises and therapeutic practices designed to facilitate the flow of qi (vital energy) (6). Historical evidence, including the colored silk painting Dao Yin diagrams unearthed from the Mawangdui Han tombs, represents the earliest known records of Qigong practices (7). Over time, forms such as Baduanjin, Wuqinxi, Liuzijue, and Yijinjing have been officially recognized and widely practiced (8). Despite their diversity, all Qigong practices share unified core principles of tiao xin (regulating the mind), tiao xi (regulating the breath), and tiao shen (regulating the body), practiced in an integrated manner (9). This combination serves as a fundamental method to enhance physical fitness, prevent illness, and cultivate overall wellbeing (10, 11).

Sharing many of these principles and benefits, Tai Chi and Qigong are regarded as the two most popular TCM practices (12). In contrast to Qigong's deep roots in TCM spanning over five millennia, Tai Chi originated approximately in the 17th century as a Chinese martial art, characterized by flowing sequences of movements coordinated with breath, serving purposes of both self-defense and health cultivation (13). Accordingly, investigators and practitioners conventionally regard the two as distinct practices (14). Nevertheless, a growing body of research worldwide has examined Tai Chi and Qigong comparatively or in combination (11). Beyond Tai Chi, Qigong is increasingly studied alongside other traditional mind-body practices from diverse cultural traditions, such as yoga (15) and meditation (16), collectively contributing to an expanding evidence base.

In recent years, the relevance of Qigong has expanded beyond its traditional geographic and cultural boundaries. Scientific studies have increasingly explored its clinical efficacy (17), physiological mechanisms (18), and integration with conventional medicine (19). Several clinical trials and systematic reviews have demonstrated its wide-ranging health benefits, including positive effects in cancer care (20), chronic obstructive pulmonary disease (21), diabetes (22), chronic pain (23), as well as in the management of stress, anxiety, and depression (24). Despite this growing body of evidence, knowledge of the global research landscape in Qigong studies, including its intellectual, social, and conceptual structures, remains fragmented, limiting insights into its trajectory and future directions.

Bibliometric analysis, as a quantitative and science-mapping approach, helps systematically outline the cumulative scientific knowledge and evolutionary dynamics of a field by rigorously processing large volumes of unstructured data (25). Unlike systematic review, which addresses specific clinical questions through qualitative synthesis of smaller, manageable datasets, bibliometric analysis is particularly suited to the breadth and heterogeneity of the Qigong literature and the present study's objective of mapping its global research landscape (26). When well conducted, it provides a comprehensive overview of the research landscape, uncovers knowledge gaps, inspires novel investigation ideas, and informs both research and clinical applications (27). Thus, employing VOSviewer, R software, and CiteSpace to visually map the knowledge evolution, collaborative networks, scholarly impact, research hotspots, and emerging clinical trends in Qigong, this study aims to equip researchers and clinicians with actionable insights to guide future research, shape health policy, and promote the integration of Qigong into evidence-informed health care.

## 2 Materials and methods

### 2.1 Data collection

The data were retrieved from the Web of Science Core Collection (WoSCC) and PubMed databases on August 18, 2025. Based on the announcement of the Health Qigong Center of the General Administration of Sport of China regarding the catalog of Health Qigong Promotion, corresponding search terms were developed (8). Detailed search strategies and selection criteria for both databases are presented in Table 1. Publication types were limited to “Article” and “Review Article” in WoSCC and “Clinical Study” in PubMed. The language was restricted to “English”, and the publication period spanned from 2005 to 2025. After excluding irrelevant and duplicate records, a total of 1,730 publications were obtained from WoSCC and 387 from PubMed. The WoSCC publications comprised 1,132 articles and 598 reviews. Among the PubMed clinical studies, randomized controlled trials (RCTs) were the predominant design (*n* = 266), followed by clinical trial protocols (*n* = 64), controlled clinical trials (*n* = 18), observational studies (*n* = 8), and other clinical trials (*n* = 31). WoSCC records were exported in plain text format, including full records and cited references, while PubMed data were downloaded in PubMed format with all available information.

**Table 1:** Search strategies and selection criteria for literature retrieved from WoSCC and PubMed.

Parameter	WoSCC	PubMed
Retrieval date	August 18, 2025	
Search field	Topic Search (TS)	Title/Abstract (tiab)
Search terms	“Qigong” OR “Qi Gong” OR “Ch'i Kung” OR “Yijinjing” OR “Yi jin jing” OR “Wuqinxi” OR “Wu qin xi” OR “Liuzijue” OR “Liu zi jue” OR “Baduanjin” OR “Ba duan jin” OR “Taiji stick” OR “Tai chi stick” OR “Daoyin” OR “Dao yin” OR “Shierduanjin” OR “Shi er duan jin” OR “Mawangdui daoyin” OR “Mawangdui daoyinshu” OR “Dawu” OR “Da wu” OR “Mingmugong” OR “Ming mu gong”	
Publication period	January 1, 2005, to August 18, 2025	
Document types	Article or Review	Clinical Study
Language	English only	
Data cleaning	Irrelevant and duplicate records were excluded	
Results	1,730 publications retrieved	387 publications retrieved
Data export	Exported in plain text format with full records and cited references	Downloaded in PubMed format with all available information

TS, Topic Search (Title, Abstract, Author Keywords, Keywords Plus).

### 2.2 Data analysis

This study primarily conducted a bibliometric analysis based on the WoSCC, widely recognized as the world's oldest, most authoritative, and extensively used database for research publications and citations (28). Moreover, PubMed, an essential source of biomedical literature, was consulted to capture clinical research focus and trends in the field, ensuring both the comprehensiveness of the dataset and the methodological rigor of the study (29).

This study employed three key software tools, R (Version 4.2.2), VOSviewer (Version 1.6.20), and CiteSpace (Version 6.4.R1), each serving distinct and complementary analytical purposes. Bibliometrix, an R-based package capable of rapid data processing and science mapping (30), was used to calculate fundamental bibliometric metrics and visualize scientific production by corresponding authors' countries. Graphical visualizations, including annual publication trends and leading sources, were generated using the ggplot2 package within RStudio. VOSviewer, a Java-based software specialized in constructing and visualizing bibliometric networks (31), was applied to map country and institutional co-authorship networks, perform source co-citation analysis, and explore keyword co-occurrence patterns. CiteSpace, which integrates mathematical modeling and statistical algorithms (32), was employed to identify clinical hotspots and emerging frontiers through its burst detection function.

Additionally, network clustering in VOSviewer was performed using the Smart Local Moving (SLM) algorithm, a modularity-based method that identifies communities by optimizing a weighted objective function. Burst detection in CiteSpace was conducted using Kleinberg's algorithm, which models an item's frequency over time as a finite-state automaton and identifies periods of significantly elevated occurrence. For network analyses, thresholds were set based on dataset size and visualization clarity: 1 publication for country co-authorship, 10 publications for institutional co-authorship, 150 citations for source co-citation, and 20 occurrences for keyword co-occurrence. Across these networks, link strength between two items refers to the number of documents in which they co-occur, and total link strength refers to the sum of all pairwise link strengths for a given item. Detailed parameters are provided in Supplementary material 1, and the original software references contain more comprehensive formulas and computational procedures (31, 33). Impact Factors (IFs) were obtained from the 2025 Journal Citation Reports (JCR), and disease classifications followed the International Statistical Classification of Diseases and Related Health Problems, 10th Revision (ICD-10).

## 3 Results

### 3.1 Overview of selected studies on Qigong

**Figure 1:**
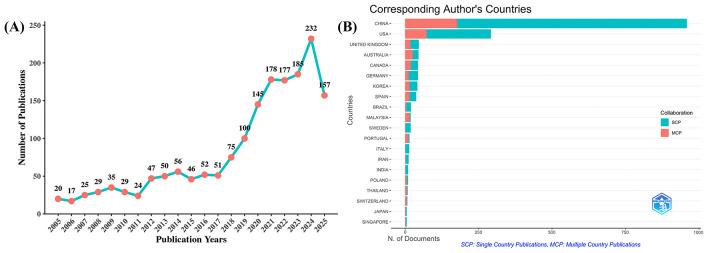
Trends in annual publication outputs on Qigong from 2005 to 2025. **(A)** Trends of annual publication outputs. **(B)** Distribution of corresponding authors' countries and cooperation. Teal bars indicate Single Country Publications (SCP) and salmon bars indicate Multiple Country Publications (MCP).

A total of 1,730 unique publications were retrieved from WoSCC after duplicates were removed. Between 2005 and 2025, the number of publications on Qigong exhibited steady growth with minor fluctuations, as shown in Figure 1A, reflecting sustained scholarly interest in the field. The annual output remained relatively modest prior to 2012, followed by a marked surge from 2018 onward, peaking at 232 articles in 2024. This growth was predominantly driven by increased output from China and the USA, which likely reflects policy prioritization and substantial governmental investment in traditional medicine. Notably, by August 18, 2025, 157 articles had already been published, indicating continued and robust research activity on Qigong.

**Figure 2:**
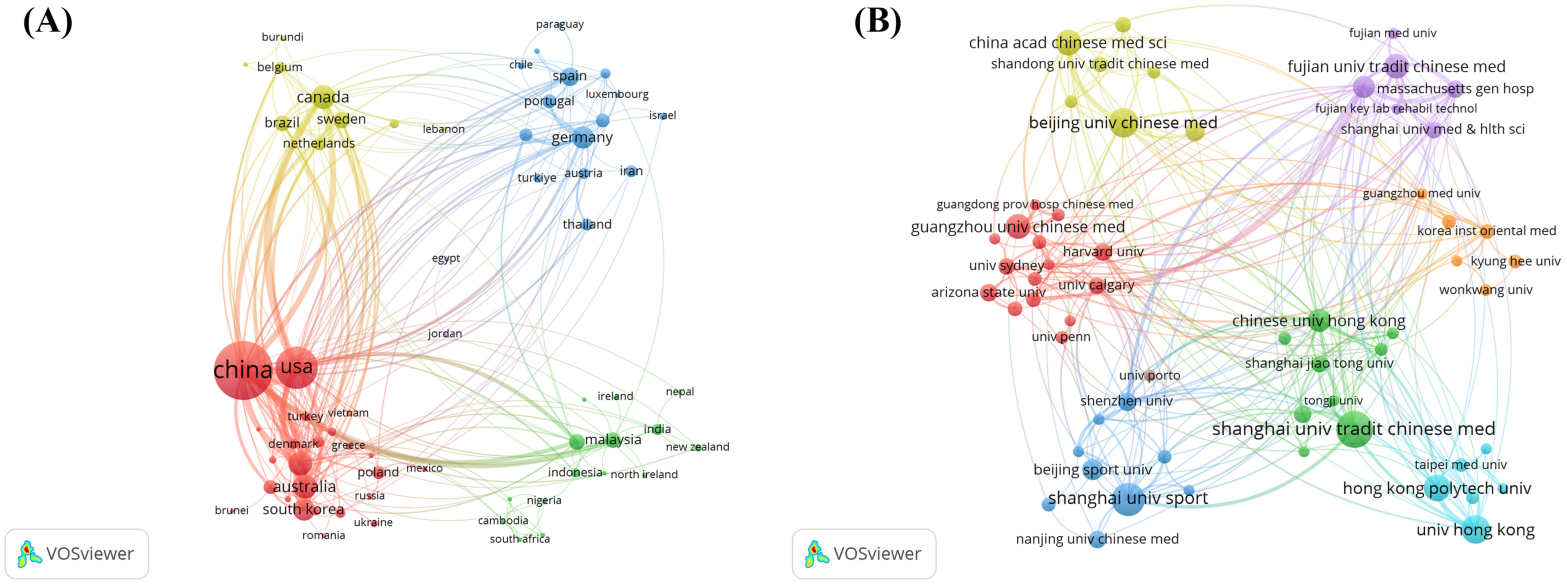
Map of countries and institutions involved in Qigong research. **(A)** Map of cooperation between different countries. **(B)** Map of cooperation between different institutions. Each node represents a country/institution; node size reflects publication volume; line thickness indicates collaboration intensity; node color denotes geographic/institutional cluster.

Analysis of corresponding authors' countries reveals that China dominates the field with 959 publications (55.4%), followed by the USA (292, 16.9%), the United Kingdom (47, 2.7%), Australia (46, 2.7%), Canada (44, 2.5%), and Germany (44, 2.5%), reflecting active global engagement with TCM. The proportion of Multiple Country Publications (MCP) is particularly high in Malaysia (78.9%), Australia (56.5%), and Canada (43.2%), indicating strong international research cooperation (Table 2, Figure 1). Moreover, as illustrated in Figure 2A, among the 66 countries analyzed, China ranks not only as a leading contributor in terms of publications but also exhibits high citations and total link strength, serving as a central hub for knowledge dissemination and global scholarly collaboration.

**Table 2:** Most relevant countries by corresponding authors of Qigong research.

Country	Articles	Freq (%)	SCP	MCP	MCP_Ratio (%)
China	959	55.4	782	177	18.5
USA	292	16.9	219	73	25.0
United Kingdom	47	2.7	29	18	38.3
Australia	46	2.7	20	26	56.5
Canada	44	2.5	25	19	43.2
Germany	44	2.5	32	12	27.3
Korea	42	2.4	27	15	35.7
Spain	38	2.2	23	15	39.5
Brazil	20	1.2	16	4	20.0
Malaysia	19	1.1	4	15	78.9

MCP, Multiple country publication; SCP, Single country publication.

As shown in Table 3, among 2,096 organizations, institutional contributions to Qigong research are predominantly concentrated in China and the USA. Shanghai University of Traditional Chinese Medicine accounts for nearly 10.9% of total publications (*n* = 188), followed by Harvard University (*n* = 152), Guangzhou University of Chinese Medicine (*n* = 114), Harvard University Medical Affiliates (*n* = 113), and the University of Hong Kong (*n* = 111). With a minimum threshold of 10 publications per organization, 59 institutions meet the criteria, and their collaboration networks are illustrated in Figure 2B. Regional clusters and partnerships suggest that institutional collaborations tend to exhibit geographic proximity. Notably, Chinese universities specializing in TCM and sports, alongside Harvard and its affiliated institutions, play authoritative and efficient roles in advancing both scholarly output and international academic exchange.

**Table 3:** Top 10 most relevant affiliations of Qigong research.

Rank	Institution	Articles
1	Shanghai University of Traditional Chinese Medicine	188
2	Harvard University	152
3	Guangzhou University of Chinese Medicine	114
4	Harvard University Medical Affiliates	113
5	University of Hong Kong	111
6	Shanghai University of Sport	104
7	Fujian University of Traditional Chinese Medicine	93
8	Beijing University of Chinese Medicine	87
9	China Academy of Chinese Medical Sciences	73
10	Harvard Medical School	70

### 3.2 Analysis of funding agencies

To characterize the funding landscape of Qigong research, funding agency data were extracted from WoSCC records using CiteSpace, with manual merging applied to consolidate variant spellings of the same source.

The top 10 funding agencies by frequency are presented in Table 4. The National Natural Science Foundation of China ranks first (*n* = 148), a vice-ministerial institution under the Ministry of Science and Technology, followed by the National Institutes of Health (*n* = 89), part of the United States Department of Health and Human Services and the nation's principal medical research agency. Other leading funders include the National Key Research and Development Program of China (*n* = 38), the National Institute for Health Research (*n* = 29) from the United Kingdom, and the National Social Science Foundation of China (*n* = 23).

**Table 4:** Top 10 funding agencies of Qigong research.

Rank	Funding agency	Count
1	National Natural Science Foundation of China	148
2	National Institutes of Health	89
3	National Key Research and Development Program of China	38
4	National Institute for Health Research	29
5	National Social Science Foundation of China	23
6	Fundamental Research Funds for the Central Universities	21
7	Fujian Provincial Rehabilitation Industrial Institution	13
8	Shanghai Key Lab of Human Performance	11
9	Korea Institute of Oriental Medicine	11
10	National Basic Research Program of China	10

Notably, the majority of these top funders operate at the national level and represent public funding sources, while the inclusion of the Fujian Provincial Rehabilitation Industrial Institution (*n* = 13) and the Shanghai Key Lab of Human Performance (*n* = 11) indicates that regional level funding also contributes to the field. The presence of funders from China, the USA, the United Kingdom, and Korea indicates that governmental interest in Qigong research extends internationally.

### 3.3 Source analysis and visualization

To identify sources with the highest publication and citation contributions in the field of Qigong, we employed the Bibliometrix package in R and generated graphical representations using ggplot2. Co-cited source analysis was further conducted using VOSviewer.

**Figure 3:**
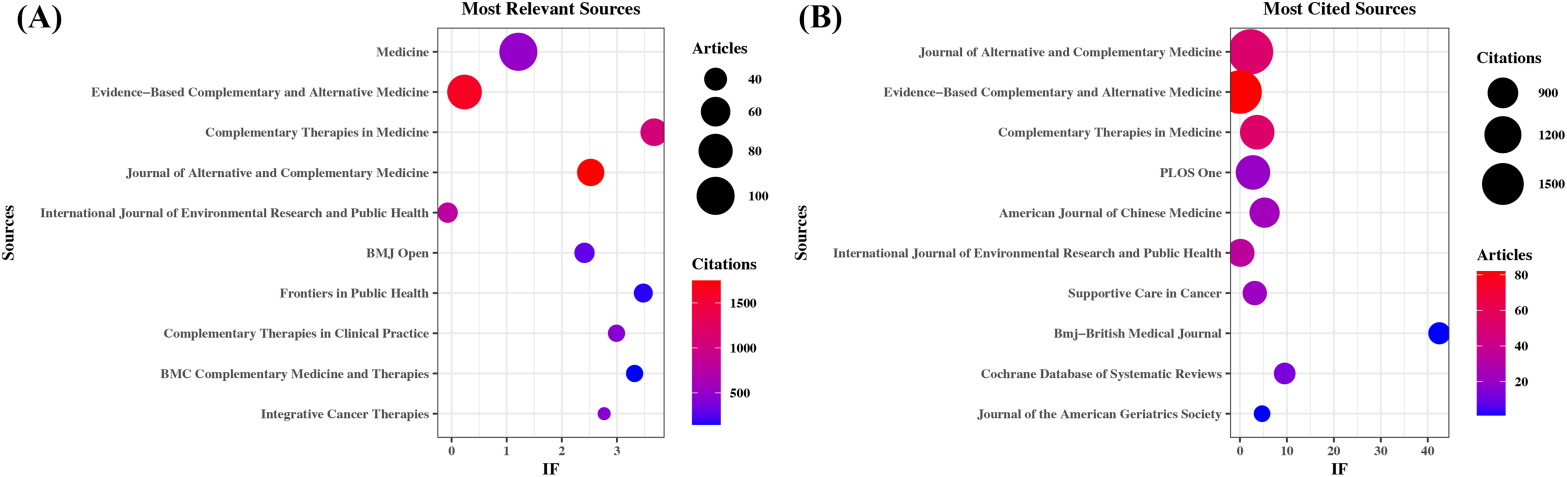
Sources with the highest article output and citation counts in Qigong research. **(A)** Most relevant sources. Bubble size indicates article count; color signifies citation count. **(B)** Most cited sources. Bubble size indicates citation count; color signifies article count.

**Figure 4:**
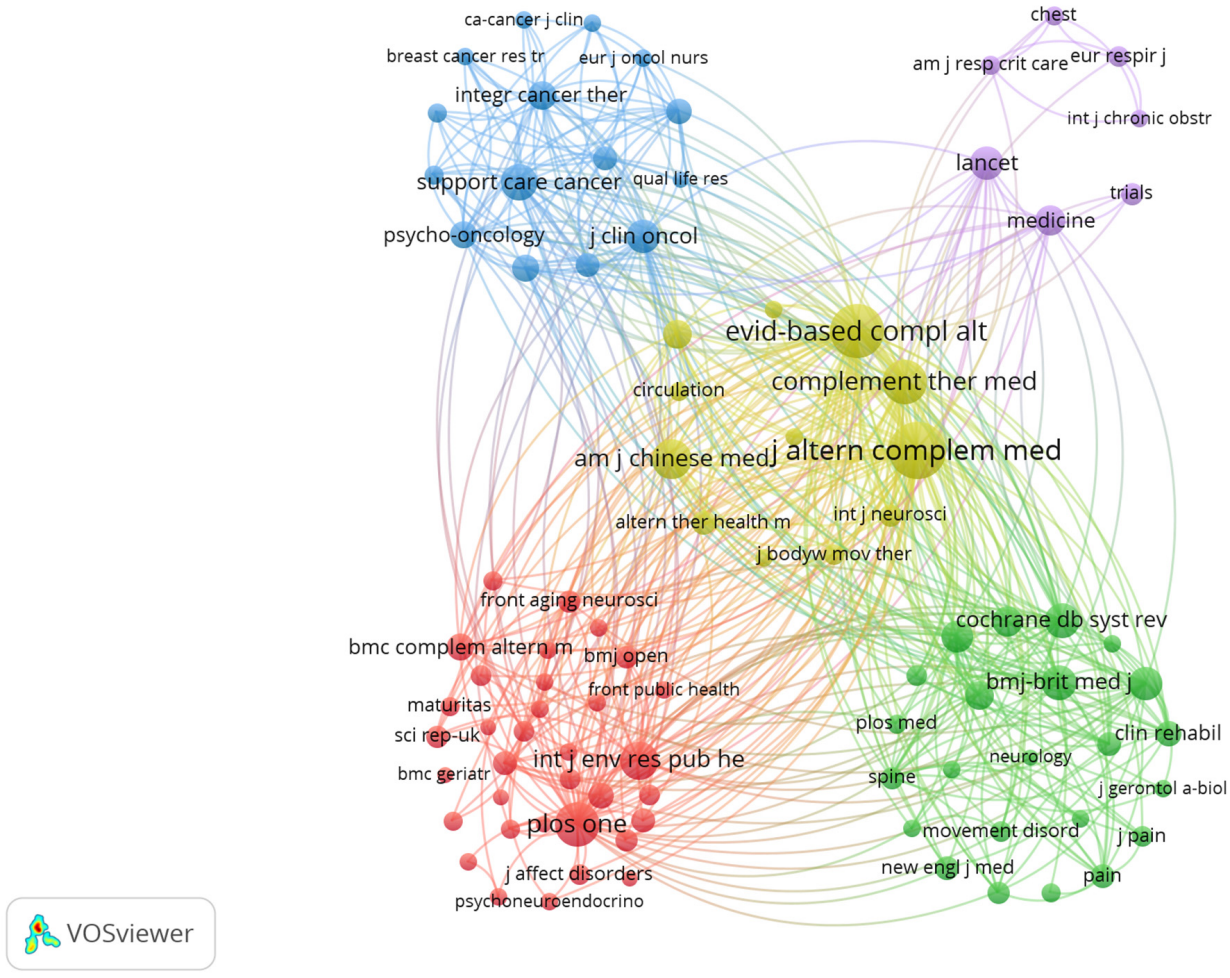
Co-cited sources related to Qigong research. Each node represents a journal; node size reflects co-citation frequency; line thickness indicates the strength of co-citation linkage between two sources; node color denotes co-citation cluster.

According to Bibliometrix, the documents are published across 550 scholarly sources. As shown in Table 5 and Figure 3A, *Medicine* (*n* = 100, IF = 1.4) ranks as the most prolific source. Next in rank are *Evidence-Based Complementary and Alternative Medicine* (*n* = 82, IF = N/A) and *Complementary Therapies in Medicine* (*n* = 53, IF = 3.5). The most frequently cited sources are presented in Table 6 and Figure 3B. *Journal of Alternative and Complementary Medicine* (1,748 citations, IF = 2.3) leads in citations, followed by *Evidence-Based Complementary and Alternative Medicine* (1,623 citations) and *Complementary Therapies in Medicine* (1,069 citations). It is worth noting that in the source co-citation map (Figure 4), these journals likewise rank as the top three in terms of total link strength, indicating their pivotal role in shaping the intellectual structure and scholarly connectivity of Qigong research.

**Table 5:** Top 10 most relevant sources of Qigong research.

Source	Articles	Cites	IF (2025)
Medicine	100	510	1.4
Evidence-Based Complementary and Alternative Medicine	82	1623	–
Complementary Therapies in Medicine	53	1069	3.5
Journal of Alternative and Complementary Medicine	52	1748	2.3
BMJ Open	35	285	2.3
International Journal of Environmental Research and Public Health	35	803	–
Frontiers in Public Health	33	168	3.4
BMC Complementary Medicine and Therapies	31	146	3.4
Complementary Therapies in Clinical Practice	31	436	3.0
Integrative Cancer Therapies	29	439	2.8

**Table 6:** Top 10 most cited sources of Qigong research.

Source	Cites	Articles	IF (2025)
Journal of Alternative and Complementary Medicine	1,748	52	2.3
Evidence-Based Complementary and Alternative Medicine	1,623	82	–
Complementary Therapies in Medicine	1,069	53	3.5
PLOS One	1067	20	2.6
American Journal of Chinese Medicine	884	24	5.5
International Journal of Environmental Research and Public Health	803	35	–
Supportive Care in Cancer	711	22	3.0
BMJ-British Medical Journal	670	1	42.7
Cochrane Database of Systematic Reviews	664	13	9.4
Journal of the American Geriatrics Society	629	1	4.5

These findings indicate that journals specializing in complementary and alternative medicine serve as the primary outlets for Qigong research in terms of both publication volume and scholarly impact. In addition, several core sources, including *BMJ Open* and *Frontiers in Public Health*, are multidisciplinary open-access journals, ensuring that Qigong research is widely accessible, transparent, and rapidly disseminated across diverse fields.

### 3.4 Analysis of references with strongest citation bursts

**Figure 5:**
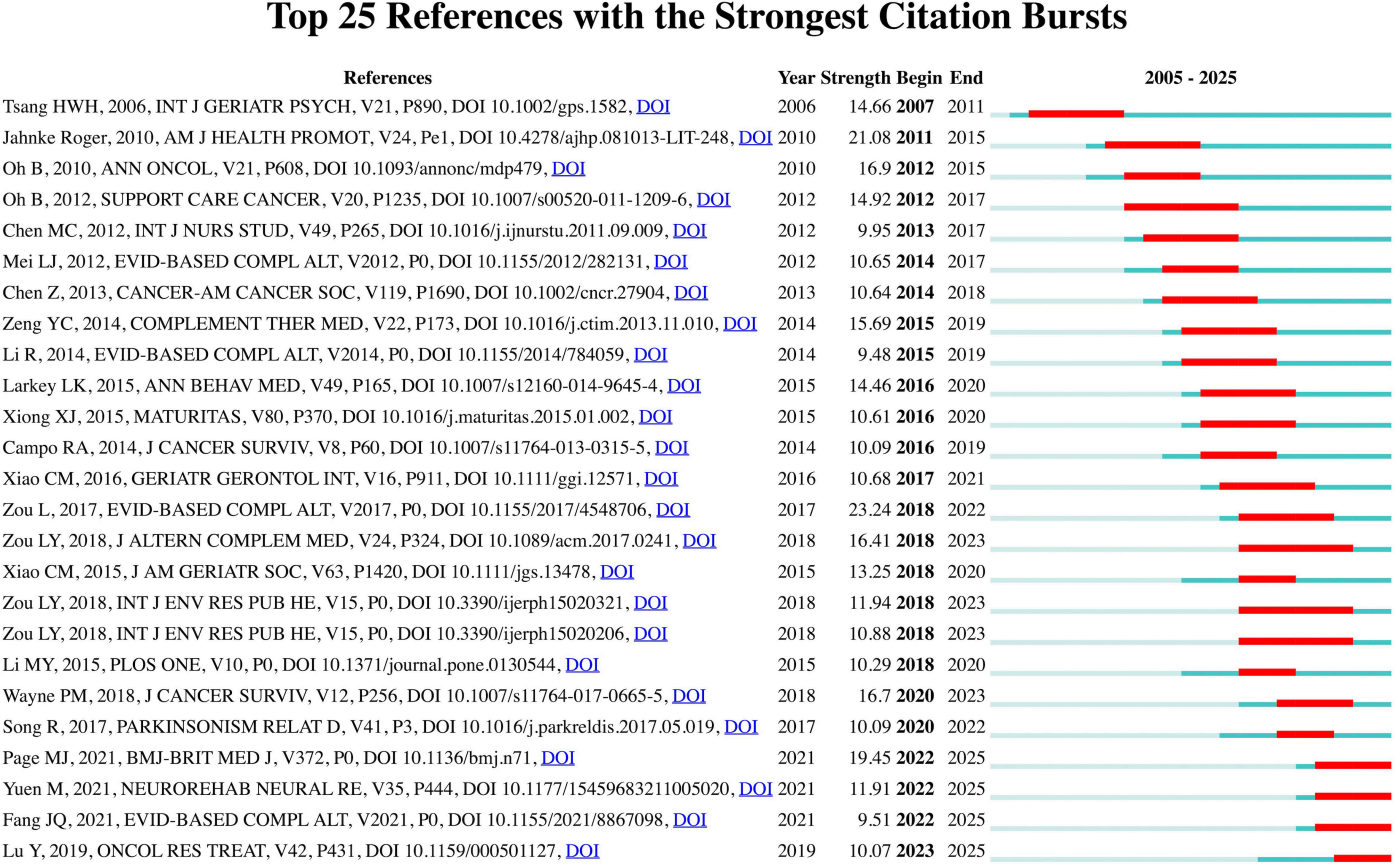
Top 25 references with the strongest citation bursts related to Qigong research.

To explore the forefront and focal areas of research on Qigong, CiteSpace was employed to identify the top 25 most significant citation bursts related to Qigong (Figure 5). Notably, the three references exhibiting the strongest citation bursts are: (1) “A Systematic Review and Meta-Analysis of Baduanjin Qigong for Health Benefits: Randomized Controlled Trials” (strength: 23.24) (34); (2) “A Comprehensive Review of Health Benefits of Qigong and Tai Chi” (strength: 21.08) (11); and (3) “The PRISMA 2020 Statement: An Updated Guideline for Reporting Systematic Reviews” (strength: 19.45) (35).

In addition, the three most recent references identified with citation bursts are: (1) “Baduanjin Qigong Improves Balance, Leg Strength, and Mobility in Individuals with Chronic Stroke: A Randomized Controlled Study (36)”; (2) “The Safety of Baduanjin Exercise: A Systematic Review (37)”; and (3) “Effect of Baduanjin Qigong Exercise on Cancer-Related Fatigue in Patients with Colorectal Cancer Undergoing Chemotherapy: A Randomized Controlled Trial (38).”

Analysis by citation burst strength reveals a strong scholarly focus on systematic reviews. The top two are systematic reviews specifically examining the health effects of Qigong-related practices, while the inclusion of the PRISMA 2020 Statement, a widely adopted guideline for reporting systematic reviews, reflects the growing emphasis on standardized and transparent methodologies for knowledge generation in this field. Analysis by recency suggests that Baduanjin represents the most current research focus, with recent references addressing this specific modality across diverse clinical contexts, including stroke rehabilitation, cancer-related fatigue, and safety evaluation.

### 3.5 Analysis of keyword co-occurrence and thematic clusters

Keywords are pivotal for summarizing the core content and mapping the knowledge structure of a field. In this study, VOSviewer was applied to analyze keywords co-occurrence in Qigong research. After merging variations, a total of 134 keywords with a minimum occurrence of 20 were retained for keyword analysis.

Table 7 lists the top 20 most frequently occurring keywords, reflecting the primary research focuses within the dataset. The most prevalent keywords were Qigong (*n* = 664), Tai Chi (*n* = 485), exercise (*n* = 438), quality-of-life (*n* = 418), and randomized controlled trials (*n* = 241), indicating a strong emphasis on clinical interventions and health outcomes associated with Qigong practice.

**Table 7:** Top 20 frequent keywords in Qigong research.

Rank	Keywords	Count
1	qigong	664
2	tai chi	485
3	exercise	438
4	quality-of-life	418
5	randomized controlled trials	241
6	baduanjin	232
7	health	206
8	older-adults	205
9	meta-analysis	204
10	physical-activity	191
11	depression	181
12	yoga	164
13	anxiety	153
14	systematic review	145
15	interventions	143
16	rehabilitation	130
17	therapy	130
18	fatigue	129
19	symptoms	126
20	management	119

**Figure 6:**
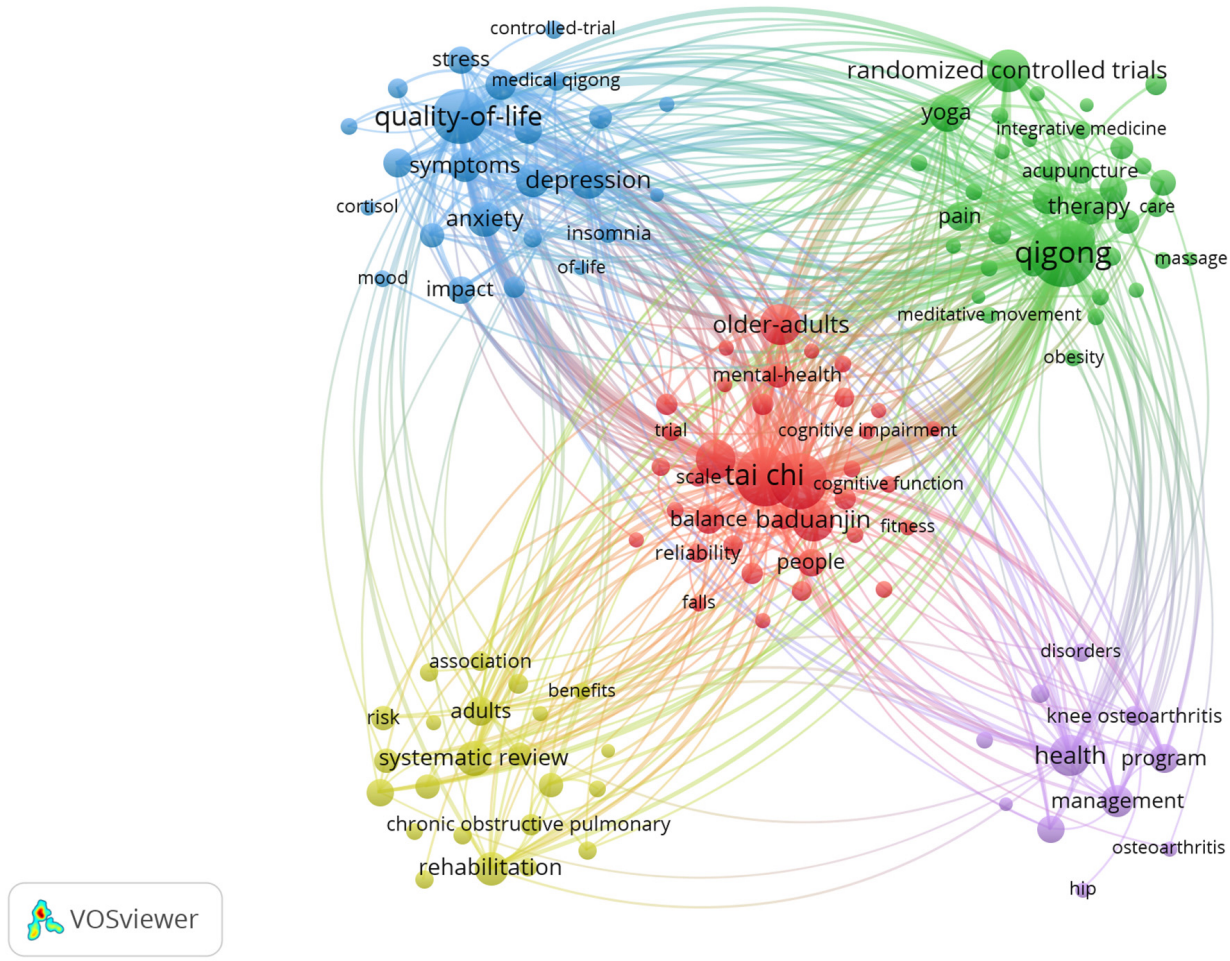
Keyword clustering map of Qigong research. Each node represents a keyword; node size reflects occurrence frequency; line thickness indicates co-occurrence strength between two keywords; node color denotes thematic cluster.

Subsequently, a keyword clustering map Figure 6 was constructed, visually highlighting five major thematic areas within the Qigong field: (1) Qigong for cognitive and physical health in older adults (red dots), comprising 38 keywords, including Baduanjin, Health Qigong, cognitive function, elderly, balance, gait, dementia, mild cognitive impairment, Parkinson's disease, stroke, and so on. The thematic emphasis focuses on the mechanisms and outcomes of Qigong and other mind-body exercises in preventing decline and maintaining or improving cognitive and motor functions among elderly populations. (2) Complementary and alternative medicine Qigong interventions for chronic conditions (green dots), comprising 37 keywords, including acupuncture, traditional Chinese medicine, complementary medicine, integrative medicine, chronic pain, hypertension, obesity, low-back-pain, and so on. This cluster highlights Qigong and broader complementary therapies for managing chronic diseases. (3) Qigong in cancer care and quality-of-life improvement (blue dots), comprising 24 keywords, including medical Qigong, breast-cancer, cancer-related fatigue, depression, anxiety, sleep quality, inflammation, quality-of-life, and so on. This cluster emphasizes the impact of Qigong in alleviating cancer-related symptoms and enhancing individual wellbeing. (4) Qigong for pulmonary rehabilitation (yellow dots), comprising 24 keywords, including chronic obstructive pulmonary disease (COPD), pulmonary rehabilitation, traditional Chinese exercise, muscle strength, systematic review, meta-analysis, and so on. This cluster focuses on evidence synthesis evaluating the benefits and risks of Qigong for pulmonary function and respiratory conditions. (5) Qigong in musculoskeletal health (purple dots), comprising 11 keywords, including knee osteoarthritis, hip, disability, disorders, program, self-efficacy, and so on. This cluster addresses the application of Qigong in managing musculoskeletal disorders and promoting functional recovery. All keywords contained in the five clusters can be found in Supplementary material 2.

### 3.6 Analysis of disease/condition-related MeSH keywords in clinical studies

A total of 679 MeSH keywords were extracted from the included 387 clinical studies on Qigong. Applying a minimum co-occurrence threshold of 5, 134 keywords met the criterion (complete list in Supplementary material 3). Focusing on disease/condition-related keywords, the most frequently occurring term is depression (*n* = 33), followed by fatigue (*n* = 31), anxiety and pulmonary disease, chronic obstructive (both *n* = 22). The top 20 disease/condition-related MeSH terms are presented in Table 8.

**Table 8:** Top 20 disease/condition-related MeSH terms in Qigong clinical studies.

Rank	MeSH label	Occurrences
1	depression	33
2	fatigue	31
3	anxiety	22
4	pulmonary disease, chronic obstructive	22
5	breast neoplasms	21
6	stress, psychological	19
7	cognitive dysfunction	18
8	neoplasms	17
9	chronic pain	15
10	sleep wake disorders	15
11	stroke	15
12	pain	14
13	parkinson disease	14
14	low back pain	12
15	neck pain	11
16	covid-19	10
17	diabetes mellitus, type 2	10
18	fibromyalgia	10
19	osteoarthritis, knee	10
20	frailty	9

Subsequently, these high-frequency terms were categorized according to the ICD-10 classification (Table 9). The most represented category is mental and behavioral disorders, including depression, anxiety, stress, cognitive dysfunction, and autistic disorder, with a subtotal of 97 occurrences. Following this, symptoms, signs, and abnormal clinical and laboratory findings, not elsewhere classified, such as fatigue, chronic pain, and dyspnea, account for 75 occurrences. Neoplasms, encompassing breast neoplasms, general neoplasms, and lung neoplasms, are also prominent (subtotal = 46). Diseases of the musculoskeletal system and connective tissue, including low back pain, neck pain, fibromyalgia, and knee osteoarthritis, follow closely (subtotal = 43). Diseases of the nervous system, encompassing sleep-wake disorders, Parkinson disease, and related conditions, total 41 occurrences. These patterns indicate that current research spans multiple diseases and health problems, suggesting that interventions targeting psychological wellbeing, cancer care, musculoskeletal management, and neurological conditions remain central topics in the clinical landscape.

**Table 9:** High-frequency disease/condition-related MeSH keywords classified by ICD-10.

ICD-10 chapter	MeSH label	Occurrences	Subtotal
II Neoplasms	breast neoplasms	21	46
	neoplasms	17	
	lung neoplasms	8	
IV Endocrine, nutritional and metabolic diseases	diabetes mellitus, type 2	10	15
	obesity	5	
V Mental and behavioral disorders	depression	33	97
	anxiety	22	
	stress, psychological	19	
	cognitive dysfunction	18	
	autistic disorder	5	
VI Diseases of the nervous system	sleep wake disorders	15	41
	parkinson disease	14	
	fatigue syndrome, chronic	7	
	sleep initiation and maintenance disorders	5	
IX Diseases of the circulatory system	stroke	15	31
	hypertension	9	
	heart failure	7	
X Diseases of the respiratory system	pulmonary disease, chronic obstructive	22	22
XIII Diseases of the musculoskeletal system and connective tissue	low back pain	12	43
	neck pain	11	
	fibromyalgia	10	
	osteoarthritis, knee	10	
XVIII Symptoms, signs and abnormal clinical and laboratory findings, not elsewhere classified	fatigue	31	75
	chronic pain	15	
	pain	14	
	frailty	9	
	dyspnea	6	
XX External causes of morbidity and mortality	accidental falls	6	6
XXI Factors influencing health status and contact with health services	burnout, professional	7	7
XXII Codes for special purposes	covid-19	10	10

### 3.7 Analysis of Qigong categories in clinical studies

To identify specific Qigong forms examined in clinical studies, author keywords from the included 387 PubMed publications were analyzed using VOSviewer. A total of 598 unique keywords were extracted. Synonyms and variant spellings were then merged, resulting in the identification of seven Qigong-specific forms.

As shown in Table 10, Baduanjin is by far the most frequently indexed form with 66 keyword occurrences, spanning a broad range of clinical domains including cancer, mental, cardiovascular, respiratory, musculoskeletal, and metabolic diseases. Liuzijue is predominantly applied in respiratory conditions such as COPD, post-surgical pulmonary rehabilitation, COVID-19, and Parkinson's respiratory dysfunction, as well as post-stroke dysarthria. Yijinjing is primarily directed toward musculoskeletal conditions such as chronic low back pain, cervical spondylopathy, and osteoporosis in postmenopausal women. Daoyin (*n* = 7), Wuqinxi (*n* = 6), and Chan-chuang Qigong (*n* = 5) appear less frequently, with Abdomen-rubbing Qigong recorded only once. Notably, several officially standardized forms, such as Shierduanjin, and Mingmugong, are not represented among the indexed keywords, suggesting their limited visibility in the clinical literature.

**Table 10:** Qigong forms identified through author keywords and their primary clinical applications in clinical studies.

Rank	Qigong form	Occurrences	Primary clinical applications
1	Baduanjin	66	Oncology, mental disorders, cardiovascular diseases, respiratory diseases, musculoskeletal disorders, metabolic diseases, etc.
2	Liuzijue	17	Respiratory diseases (COPD, pulmonary rehabilitation, covid-19, Parkinson's respiratory dysfunction, post-stroke dysarthria)
3	Yijinjing	11	Musculoskeletal disorders (low back pain, neck pain,osteoporosis)
4	Daoyin	7	Respiratory diseases, chronic pain
5	Wuqinxi	6	Knee osteoarthritis
6	Chan-chuang Qigong	5	Oncology
7	Abdomen-rubbing Qigong	1	Chronic insomnia

Furthermore, the geographic origin of each keyword occurrence was traced based on the institutional affiliation metadata of the indexed records. The results reveal that studies examining specific Qigong forms are overwhelmingly concentrated within the Greater China region. The principal exception is Baduanjin, which shows limited international diffusion, with isolated occurrences from institutions in Sweden, Turkey, Egypt, and Australia. Liuzijue likewise demonstrates minimal international presence, with only a single occurrence identified from the USA. These findings suggest that the international dissemination of specific Qigong forms remains relatively limited, and that the corresponding global clinical evidence base is predominantly shaped by Chinese scholarship.

### 3.8 Analysis of recent keyword trends in clinical studies

**Figure 7:**
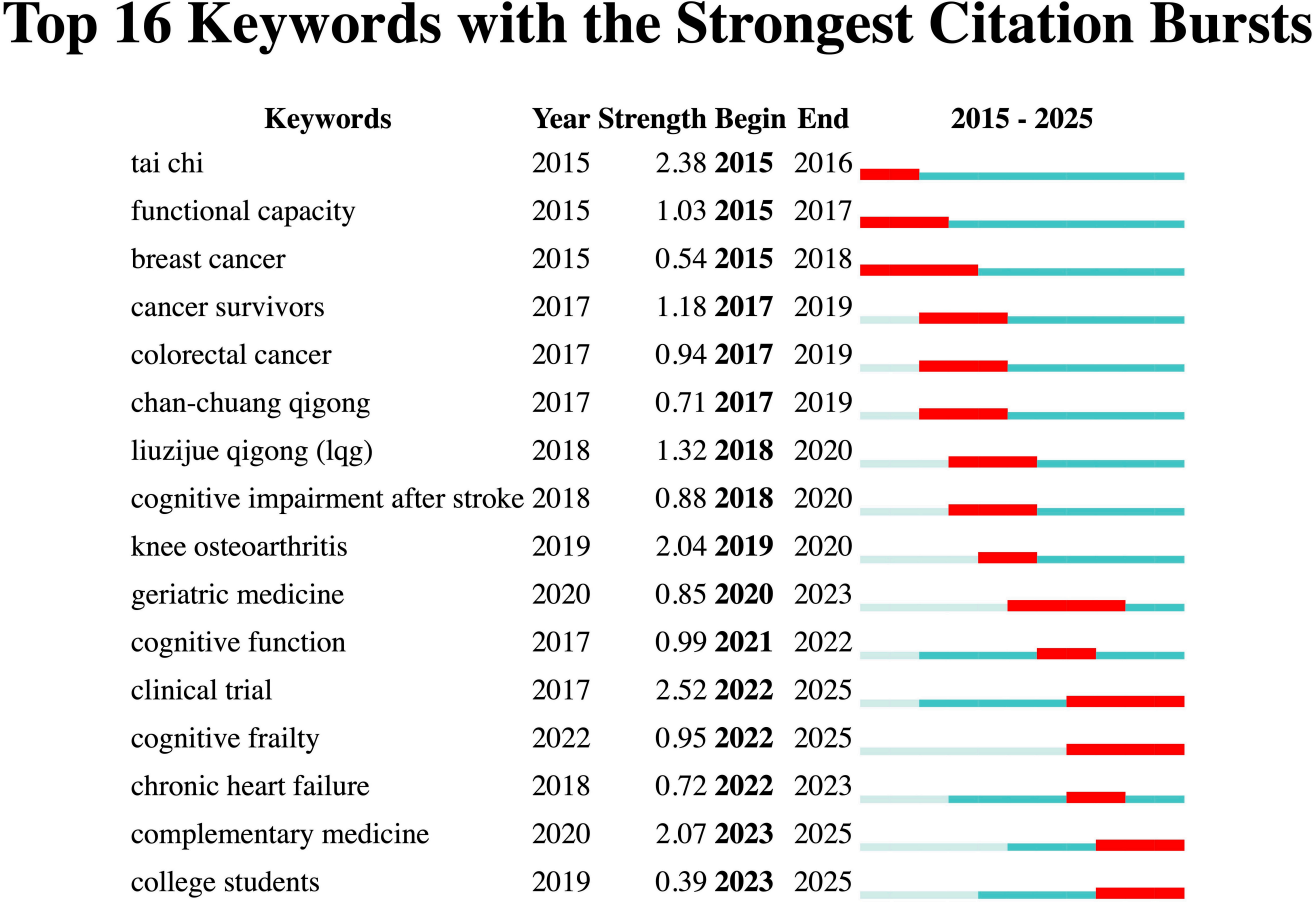
Top 16 keywords with the strongest citation bursts in Qigong clinical studies.

To capture clinically relevant dynamics and highlight emerging trends, we analyzed keyword evolution in Qigong clinical studies using CiteSpace, focusing on the last decade (2015–2025), when research activity became more substantial and thematically diverse. Figure 7 displays the 16 keywords with the strongest citation bursts, providing a snapshot of the evolving focus in Qigong clinical research.

From 2015 to 2017, studies centered on cancer-related topics, including breast cancer, cancer survivors, and colorectal cancer. Tai chi was frequently mentioned alongside Qigong as part of mind–body therapies, while specific forms such as chan-chuang and liuzijue also appeared, signaling growing diversity in practice. Between 2018 and 2020, the emphasis shifted toward rehabilitation and chronic conditions, with keywords such as cognitive impairment after stroke, knee osteoarthritis, and geriatric medicine, highlighting research in older adult populations. Since 2021, investigations have increasingly addressed cognitive health, as reflected in terms like cognitive function and cognitive frailty, underscoring a focus on maintaining or improving brain function. Notably, college students (2023–2025) emerged as one of the most recently identified keywords with the strongest citation bursts, representing a current research frontier in interventions targeting younger populations.

## 4 Discussion

### 4.1 General information

To better capture the global research perspectives on Qigong, we conducted a bibliometric analysis and data visualization based on 1,730 unique publications from WoSCC and 387 from PubMed, spanning 2005–2025. The results reveal a steady growth with minor fluctuations, followed by a marked surge from 2018 onward, peaking at 232 articles in 2024, with research activity remaining robust to date. This increase may be partly attributable to several global and national developments: the Declaration of Astana 2018, which emphasized the integration of traditional medicine knowledge and technologies into primary health care (3); the WHO Global Report on Traditional and Complementary Medicine 2019, which recognized traditional and complementary medicine as an important yet often underutilized health resource (39); and the ongoing initiatives of the Chinese Health Qigong Administrative Center, which has consolidated the cultural foundation of Health Qigong through strategic action plans, guideline development, international collaboration, and the establishment of exercise prescriptions tailored for clinical and public health applications (40, 41).

At the country level, this growth in publications has been predominantly driven by China and the USA. China, recognized as the birthplace of Qigong, takes the lead with 959 publications, followed by the USA with 292 publications, together representing over 72% of total output. Seven of the ten most prolific institutions are located in China, while Chinese universities specializing in TCM and sports, alongside Harvard and its affiliates, not only produce substantial scholarly output but also serve as influential hubs that promote global academic exchange and collaboration. This pattern is further corroborated by the funding landscape. The National Natural Science Foundation of China (*n* = 148) and the National Institutes of Health of the USA (*n* = 89) emerge as the top funding agencies, reflecting a close alignment between publication leadership and governmental investment. Although the broader landscape indicates meaningful global engagement, the overall concentration of contributing countries, institutions, and funding sources predominantly within China and the USA nonetheless warrants attention. Such geographic concentration may introduce topical bias, potentially skewing research priorities toward conditions and populations most relevant to these regions, while limiting the generalizability of findings to other cultural and healthcare contexts.

In the source analysis, *Medicine* emerges as the most prolific journal, while the *Journal of Alternative and Complementary Medicine* stands out as the most frequently cited, both followed by *Evidence-Based Complementary and Alternative Medicine* and *Complementary Therapies in Medicine*. Notably, the concentration of sources with relatively modest impact factors is not unique to Qigong, but reflects a broader pattern across the traditional, complementary, alternative and integrative medicine (TCAIM) literature (42). This pattern is partly attributable to a fundamental methodological tension between the holistic and individualized nature of Qigong practice and the reductionist frameworks that underpin mainstream biomedical research, posing ongoing challenges to its broader dissemination and integration into clinical practice (43). To address these barriers, future research could adopt multidisciplinary approaches that integrate biomedical, chemical, and computational technology (44). Concurrently, standardization efforts could strengthen adherence to established reporting guidelines, such as CONSORT for clinical trials (45) and PRISMA for systematic reviews (35), while adapting research protocols to accommodate the diverse cultural contexts and preferences (43). Furthermore, incorporating principles of implementation science offers innovative methodologies for translating evidence-based Qigong interventions into real-world practice settings (46).

Encouragingly, several core sources in this field are multidisciplinary open-access journals, which promote the dissemination and visibility of Qigong research. Building on this, the broader integration of open science practices could further enhance research transparency and reproducibility, and address ongoing concerns regarding the efficacy and safety of Qigong interventions in practice (43). Overall, with the international community increasingly promoting traditional medicine at the forefront of global health care strategies, future studies are expected to expand across disciplines and cultures, foster innovative integration with conventional medicine, and contribute to the transformation of global health and wellbeing (4, 47).

### 4.2 Research hotspots

The keyword frequency analysis reveals that Tai Chi ranks second, with yoga also in the list, suggesting that Qigong research is embedded within a broader mind-body intervention landscape. This may partly reflect the value of integrating Qigong with related practices into comparative or combined frameworks to enhance statistical power and generate broader insights, while practice-specific investigations are also warranted for capturing more targeted evidence (48). A comprehensive analysis of references, keyword frequencies, and clustering patterns reveals several potential research hotspots in Qigong. Current studies primarily concentrate on three key areas.

#### 4.2.1 Qigong for cognitive and physical health in geriatric populations, highlighting the clinical efficacy, underlying mechanisms, and safety considerations

Based on our analysis of the existing literature, Qigong for cognitive and physical health in geriatric populations has emerged as a prominent research hotspot, with growing evidence highlighting its clinical efficacy, underlying mechanisms, and safety profile. Systematic reviews and randomized controlled trials consistently demonstrate that Qigong improves physical functions such as mobility, balance, and coordination, thereby reducing fall and fracture risk among sedentary older adults (49, 50). Beyond physical benefits, long-term interventions have shown significant improvements in global cognition, memory, visuospatial abilities, and language, with sleep quality emerging as an important moderator of cognitive outcomes (51, 52). Mechanistic study further suggests that Qigong promotes neuroplasticity by modulating brain connectivity, increasing brain volume, and regulating neurotrophic and inflammatory pathways (53). Evidence also indicates that Qigong improves hypothalamic–pituitary–adrenal axis regulation, lowering daytime cortisol levels and attenuating stress responses, which may facilitate successful aging (54). Importantly, across diverse settings, including rehabilitation units (55), community-based programs (56), and COVID-19 recovery contexts (57), Qigong has been reported as feasible, well-accepted, and safe, with no major adverse events observed. Collectively, these findings resonate with the WHO's stance that traditional medicine represents a valuable resource for addressing the complex health needs of aging populations (39), positioning Qigong as a promising mind–body therapy to support both cognitive and physical function in older adults.

#### 4.2.2 Qigong for disease management, including cancer care and chronic conditions, with distinct clusters in pulmonary rehabilitation and musculoskeletal health

Research has also concentrated on the use of Qigong in the management of chronic conditions that are costly to society, as well as more life-threatening diseases such as cancer. In cancer care, systematic reviews demonstrate that Qigong can improve immune function, reduce cortisol levels, and alleviate fatigue (58). Importantly, Qigong has been shown to be safe even for frail patients, supporting its role as a feasible mind–body intervention in supportive oncology care (59). For chronic conditions, growing evidence highlights its broad psychophysiological benefits. Qigong has been associated with improvements in immune markers, lung function, cardiac output, and reductions in cholesterol, blood pressure, and depressive symptoms (60). Specific styles, such as Liuzijue (21) and Wuqinxi (61), show promising effects in pulmonary rehabilitation for COPD, enhancing exercise tolerance, alleviating dyspnea, and improving nutrition status. Similarly, trials on musculoskeletal health reveal that Wuqinxi (62) and Baduanjin (63) are effective in managing knee osteoarthritis, outperforming conventional physical therapy in reducing pain, stiffness, and functional impairment. Collectively, these findings underscore Qigong's potential as a cost-effective and safe adjunctive therapy. Nevertheless, large-scale, long-term trials are needed to establish robust evidence for its integration into mainstream evidence-based healthcare systems.

#### 4.2.3 Qigong for quality-of-life enhancement across the lifespan

Qigong for quality-of-life (QoL) enhancement across diverse populations has emerged as a prominent research hotspot. According to the WHO, QoL encompasses an individual's perception of their position in life within cultural and value frameworks, including satisfaction with self, living conditions, personal relationships, and emotional states such as anxiety, depression, or despair (64). Among older adults, a simplified, easy-to-replicate Tai Chi and Qigong program implemented across 18 sites in the USA demonstrated significant reductions in perceived stress, improvements in sleep quality, and increased vitality, with high adherence and enjoyment reported (65). Notably, for socially isolated “hidden elderly,” Qigong may strengthen social networks and enhance psychosocial wellbeing when supported by elderly neighborhood volunteers (66). In middle-aged adults, an 8-week Qigong intervention lowered cortisol and blood pressure, contributing to stress reduction, emotional regulation, and enhanced mind–body wellbeing (67). Additionally, among students, scheduled Qigong practice shows potential to alleviate psychological distress, reduce stress, and enhance self-perception, suggesting a practical approach to promote wellbeing in school settings (68). Taken together, these findings highlight Qigong's versatility for enhancing QoL across the lifespan.

### 4.3 Development trends of clinical studies

Based on the analysis of disease/condition-related MeSH keywords in clinical studies, depression, fatigue, anxiety, and COPD are the most frequently investigated conditions, while mental and behavioral disorders, neoplasms, and diseases of the musculoskeletal and nervous system are the most represented categories. These patterns align with the previously identified research hotspots and highlight Qigong's considerable potential in addressing a wide spectrum of diseases and health-related symptoms.

With respect to intervention types, Baduanjin stands out as by far the most extensively examined form of Qigong, with abundant evidence supporting its benefits across diverse age groups and clinical populations (69). Multiple systematic reviews have begun to identify optimal intervention parameters for Baduanjin in specific clinical contexts. For cardiac rehabilitation after percutaneous coronary intervention, intervention durations of 1–3 months yielded greater improvements in left ventricular ejection fraction, whereas durations exceeding 3 months were more beneficial for six-minute walk distance (70). For type 2 diabetes mellitus, three sessions per week, each lasting 40–45 min, with an intervention duration of 24–48 weeks, may represent the optimal parameters for improving glucose and lipid metabolism (71).

By contrast, Liuzijue and Yijinjing appear with moderate frequency, while Daoyin, Wuqinxi, and Chan-chuang Qigong remain less explored. Several other Qigong forms appear only minimally or not at all represented. Notably, Liuzijue and Yijinjing have begun to show distinct disease-specific application profiles that align with their practice-specific characteristics. Liuzijue, which centers on respiratory vocalization and breath regulation, shows a concentration in respiratory diseases, with a network meta-analysis further confirming its superiority over other Qigong forms in improving pulmonary function among stable COPD patients (72). Yijinjing, which emphasizes tendon and bone stretching, is correspondingly concentrated in musculoskeletal conditions. However, the intervention durations across these studies vary considerably, ranging from 2 weeks to 6 months for Liuzijue and from 3 weeks to 6 months for Yijinjing, and long-term follow-up assessments remain scarce. More methodologically rigorous studies are needed to determine the optimal intervention parameters and evaluate the prognosis of these Qigong forms in their respective clinical applications.

The distributional pattern likely reflects the standardization initiatives undertaken since 2001, through which the Health Qigong Administrative Center codified Baduanjin, Liuzijue, Yijinjing, and Wuqinxi with detailed historical origins, movement sequences, key points, common errors, corrective guidance, and functional rationale (8). As such standardization may lower methodological barriers to clinical investigation, future research would benefit from developing authoritative protocols for a broader range of Qigong forms to expand the evidence base.Geographic analysis further reveals that research on specific Qigong forms is predominantly concentrated in the Greater China region. Baduanjin stands as the principal exception, with isolated studies from institutions in Sweden, Turkey, Egypt, and Australia, while Liuzijue appears in a single study from the United States. Given the cultural specificity inherent in Qigong form nomenclature, the clinical evidence for individual forms derives almost exclusively from Chinese scholarship. Thus, the generalizability of these findings to diverse cultural and healthcare contexts requires further verification. Notably, the Australian study originates from the Chinese Medicine Center, which is a collaborative institutional partnership between Western Sydney University and Beijing University of Chinese Medicine (73). This example suggests that formal international collaborations may serve as an important pathway for the global dissemination and cross-cultural validation of specific Qigong practices.

In recent clinical research, studies have predominantly addressed life-threatening conditions, especially cancer-related topics, and subsequently shifted toward chronic disease management, with notable emphasis on geriatric medicine and cognitive health in the context of global population aging. Since 2023, an emerging frontier in Qigong clinical studies has focused on college students, a population experiencing declining physical fitness and rising mental health burdens driven by academic stress and sedentary lifestyles. Multiple randomized controlled trials have demonstrated the effectiveness of Baduanjin in improving body mass, body composition (34), and various functional outcomes including cardiovascular fitness, flexibility, and balance (74). Beyond physical outcomes, Qigong interventions have also shown significant benefits for psychological health, including reductions in anxiety and depression alongside improvements in heart rate variability (75, 76). Sleep disturbances represent another emerging target in this population, with ongoing trials investigating the neurophysiological mechanisms through which Qigong may alleviate insomnia (77). More recently, the YoungFitT project has extended this line of inquiry by integrating Qigong with immersive virtual reality (VR), reflecting growing interest in leveraging technology to enhance motivation, engagement, and adherence in health-promoting practices, while highlighting the feasibility of integrating Qigong with VR environments to support psychological wellbeing and cognitive function in young adults (78, 79).

Notably, this emerging focus on younger populations resonates with national health initiatives in China, which have formalized age-specific Qigong programs, such as Mingmugong (adolescent version) and the Campus Wuqinxi (elementary, middle, and high school versions) (8). These practices are carefully tailored to the physiological and psychological characteristics of different age groups. The adolescent version of Mingmugong emphasizes ocular benefits, consisting of only six movements practiced within eight minutes, accompanied by specially composed instructional music to facilitate adoption in primary and secondary schools (80). Similarly, Campus Wuqinxi has been tailored to developmental stages: from fostering attention and correcting posture in children aged 6–11 through playful and engaging practice, to enhancing neuromuscular coordination and interhemispheric balance in adolescents aged 12–15 via symmetric and lower-limb–focused movements, and finally to improving cardiopulmonary function and strengthening muscular capacity in older teenagers aged 16–19 through multi-directional stretching, folding, and rotation aerobic exercise (80, 81). Future studies could systematically evaluate these programs using rigorous trial designs, substantiating their clinical efficacy and highlighting the public health value of Qigong for younger populations.

### 4.4 Limitations and considerations

This study provides a systematic and replicable bibliometric framework, offering a reliable reference for future investigations. Nevertheless, several limitations should be noted. First, the analysis was confined to publications indexed in WoSCC and PubMed. Although these databases are authoritative and widely recognized, this restriction may have introduced selection bias and resulted in the omission of relevant studies available in other sources (28, 82). Second, only English-language publications from the past two decades were included, potentially excluding valuable non-English studies. Additionally, due to the cultural specificity of Qigong terminology, unconventional transliterations may not have been fully captured, potentially limiting the comprehensiveness of the findings. Third, although ICD-11 introduces a pioneering supplementary chapter (Chapter 26) for traditional medicine conditions (83), the diseases/conditions analyzed in this study could not be fully coded within this framework. To ensure consistency, we adopted the old ICD-10 system, though this limits recognition of distinctive traditional medicine features like Spleen Qi Deficiency and Liver Qi Stagnation. Future studies, as ICD-11 continues to be refined, may apply this framework to achieve a more nuanced and contextually appropriate categorization of Qigong research. Fourth, as is inherent to bibliometric analysis, no quality assessment of the included studies was conducted. Quantitative estimates derived from bibliometric analyses cannot substitute for definitive, undifferentiated statements about scientific quality (84). Consequently, the findings of this study should be interpreted as indicators of research hotspots and emerging frontiers, rather than as assessments of the clinical evidence quality of the included literature.

The present study also highlights several directions for the advancement of bibliometric methodologies. Current bibliometric frameworks excel at mapping macro-level landscape, yet cross-tabulation investigations such as keyword by country and journal impact factor by country remain largely underexplored. These analyses would be valuable for deeper characterization of a research field, while dedicated support for such operations remains underdeveloped in most existing bibliometric tools. This challenge is further compounded by inconsistent and incomplete field coverage in databases. Certain metadata fields, such as funding agency and institutional affiliation, are inconsistently populated, limiting automated attribution across records. This is particularly pronounced in PubMed, where fewer structured fields are available, constraining the range of units amenable to analysis. On the software side, continued development of cross-tabulation functionality, automated data cleaning, and richer visualization capabilities would enhance the analytical depth. On the database side, future development would benefit from more consistent metadata standards and the expansion of standardized fields, such as intervention types and comparator characteristics where applicable, to enable richer and more granular indexing. Together, these advances would enable researchers and clinicians to move beyond broad pattern recognition toward deeper and more comprehensive investigation of the research landscape.

## 5 Conclusion

Our bibliometric analysis provides a comprehensive overview of the research landscape on Qigong over the past two decades, highlighting its knowledge structures, research hotspots, and emerging clinical trends. China and the USA lead in research productivity, with their institutions serving as central hubs for global academic collaboration, illustrating a dynamic of broad international participation with concentrated scholarly leadership rooted in Qigong's cultural and historical foundations. Qigong research primarily focuses on cognitive and physical health in geriatric populations, disease management for cancer care and chronic conditions, and quality-of-life enhancement across the lifespan, with recent clinical studies revealing a trajectory toward disease diversification, modality specialization, and population expansion. Research largely prioritizes a small subset of Qigong forms, with Baduanjin being the most studied, while youth-adapted practices such as Mingmugong and Campus Wuqinxi, though rarely investigated, could be explored to advance the frontier of promoting health in younger populations. These findings outline a roadmap for future investigations aimed at strengthening evidence and international collaboration to integrate Qigong into global evidence-based health care, bridging inheritance and innovation to advance holistic health and wellbeing for all.

## Data Availability

The original contributions presented in the study are included in the article/Supplementary material, further inquiries can be directed to the corresponding author.
