# Supplementary material for: Global perspectives and clinical trends in Qigong research: a bibliometric and visual analysis (2005–2025)
Source: Front Med (Lausanne). 2026 May 7;13:1707980. doi: 10.3389/fmed.2026.1707980 (PMC13190511; doi:10.3389/fmed.2026.1707980)
Supplement: Supplementary file 1 [file Data_Sheet_1.pdf]

Supplementary Material

This supplementary material provides detailed configuration parameters for all bibliometric analyses conducted in this study using VOSviewer (Version 1.6.20) and CiteSpace (Version 6.4.R1).

Four types of bibliometric networks were constructed using VOSviewer: country co-authorship, institutional co-authorship, source co-citation, and keyword co-occurrence,. For all analyses, Full counting was adopted as the counting method and association strength as the normalization approach. The similarity s_{ij} between two items i and j is calculated as:

$$s_{ij} = \frac{c_{ij}}{w_i w_j}$$

where c_{ij} denotes the number of co-occurrences of items i and j, and w_i and w_j denote the total number of occurrences (or co-occurrences) of items i and j, respectively. This measure is proportional to the ratio of the observed co-occurrences to the expected co-occurrences under statistical independence, thereby correcting for differences in item frequency. Network clustering was performed using the Smart Local Moving (SLM) algorithm with a resolution parameter of 1.00 (default). The resolution parameter γ governs cluster granularity: higher values yield more and smaller clusters, while lower values produce fewer and larger clusters.

Two burst detection analyses were conducted: citation burst detection based on WoSCC data (2005–2025) and keyword burst detection based on PubMed clinical studies (2015–2025). Both analyses applied Kleinberg's burst detection algorithm, which models an item's frequency over time using a finite-state automaton and detects intervals of significantly elevated occurrence. More detailed formulas and computational procedures are described in the original software papers [1, 2].

SUPPLEMENTARY TABLES

Table S1. VOSviewer parameters for country co-authorship network.

Parameter	Setting
Unit of analysis	Countries
Counting method	Full counting
Minimum documents threshold	1 publication
Countries retained	66
Normalization method	Association strength
Link strength	Number of documents co-authored by researchers from two countries
Total link strength	Sum of all pairwise link strengths for a given country
Clustering algorithm	Smart Local Moving (SLM), modularity-based
Resolution parameter	1.00 (default)

Table S2. VOSviewer parameters for institutional co-authorship network.

Parameter	Setting
Unit of analysis	Organizations
Counting method	Full counting
Minimum documents threshold	10 publications
Institutions retained	59
Normalization method	Association strength
Link strength	Number of documents co-authored by researchers from two institutions
Total link strength	Sum of all pairwise link strengths for a given institution
Clustering algorithm	Smart Local Moving (SLM), modularity-based
Resolution parameter	1.00 (default)

Table S3. VOSviewer parameters for source co-citation analysis.

Parameter	Setting
Unit of analysis	Cited sources (journals)
Counting method	Full counting
Minimum citations threshold	150 citations
Sources retained	91
Normalization method	Association strength
Link strength	Number of documents in which two journals are co-cited
Total link strength	Sum of all pairwise co-citation link strengths for a given journal
Clustering algorithm	Smart Local Moving (SLM), modularity-based
Resolution parameter	1.00 (default)

Table S4. VOSviewer parameters for keyword co-occurrence analysis.

Parameter	Setting
Unit of analysis	Keywords
Counting method	Full counting
Minimum occurrence threshold	20 occurrences
Keywords retained	134
Normalization method	Association strength
Link strength	Number of documents in which two keywords co-occur simultaneously
Total link strength	Sum of all pairwise link strengths for a given keyword
Clustering algorithm	Smart Local Moving (SLM), modularity-based
Resolution parameter	1.00 (default)

Table S5. CiteSpace parameters for citation burst detection (WoSCC).

Parameter	Setting
Data source	Web of Science Core Collection
Time span	2005–2025
Time slicing	1 year per slice
Node type	Reference
Node selection	g-index
Top-k per slice	25
Pruning	None
Burst detection	Kleinberg’s algorithm
Bursts reported	Top 25

Table S6. CiteSpace parameters for keyword burst detection (PubMed).

Parameter	Setting
Data source	PubMed
Time span	2015–2025
Time slicing	1 year per slice
Node type	Keyword
Node selection	g-index
Top-k per slice	25
Pruning	None
Burst detection	Kleinberg’s algorithm
Bursts reported	Top 16

REFERENCES

[1]Chaomei Chen. Citespace ii: Detecting and visualizing emerging trends and transient patterns in scientific literature. *Journal of the American Society for information Science and Technology*, 57(3):359–377, 2006.

[2]Nees Van Eck and Ludo Waltman. Software survey: Vosviewer, a computer program for bibliometric mapping. *scientometrics*, 84(2):523–538, 2010.
